# A new glycosidic flavonoid from *Jwarhar mahakashay* (antipyretic) Ayurvedic preparation

**DOI:** 10.4103/0974-7788.64401

**Published:** 2010

**Authors:** Mradu Gupta, B. P. Shaw, A. Mukherjee

**Affiliations:** *Department of Dravyaguna, Institute of Post Graduate Ayurvedic Education and Research, 294/3/1, A.P.C. Road, Kolkata – 700 009, India*; 1 *Department of Kayachikitsa, Institute of Post Graduate Ayurvedic Education and Research, 294/3/1, A.P.C. Road, Kolkata – 700 009, India*; 2 *Department of Chemical Technology, Calcutta University, 92, A.P.C. Road, Kolkata – 700 009, India*

**Keywords:** Antipyretic, chromatography, spectroscopy, polyherbal preparation, *Jwarahar mahakashay*

## Abstract

The aqueous extract of *Jwarhar mahakashay* Ayurvedic preparation (from the roots of *Hemidesmus indicus* R. Br., *Rubia cordifolia* L., Cissampelos pareira L.; fruits of *Terminalia chebula* Retz., *Emblica officinalis* Gaertn., *Terminalia bellirica* Roxb., *Vitis vinifera* L., *Grewia asiatica* L., *Salvadora persica* L. and granules of *Saccharum officinarum* L.) has been used as a traditional antipyretic. Experimental studies confirmed its antipyretic–analgesic effect with very low ulcerogenicity and toxicity. Flavonoids, glycosides and tannins were later found to be present in the extract. Detailed chemical investigations were undertaken after hydrolysis of extract using spectroscopic and chromatography methods to determine its active chemical constituent. UV-Visible spectroscopy showed absorbance maxima at 220 and 276 nm, while fourier transform infra-red investigations indicated an end carboxylic O–H structure at 2940 cm^−1^ suggesting the presence of glycoside-linked flavonoids. Thin layer chromatography and high performance liquid chromatography also confirmed the possibility of at least one major and two minor compounds in this abstract. Detailed examination using gas chromatography-mass spectrometry led to the identification of the principal component as 2-(1-oxopropyl)-benzoic acid, which is quite similar to the active compound found in the standard drug Aspirin (2-acetyl-oxybenzoic acid).

## INTRODUCTION

Traditional Indian systems of medicine such as Ayurveda are based on holistic treatment of diseases primarily relying on natural herbal drugs. Most Ayurvedic preparations are polyherbal in nature to take care of the multiple components of disease conditions. The group of antipyretic drugs has been defined in *Jwarahar Mahakashay Sutra Sthana* of Charak Samhita.[1] This *Jwarhar mahakashay* group of antipyretic drugs includes *Sariva* (*Hemidesmus indicus* R. Br.), *Manjistha* (*Rubia cordifolia* L.), *Patha* (*Cissampelos pareira* L.), *Haritaki* (*Terminalia chebula* Retz.), *Amala* (*Phyllanthus emblica* Gaertn.), *Vibhitak* (*Terminalia bellirica* Roxb.), *Draksha* (*Vitis vinifera* L), *Parushak* (*Grewia asiatica* L), *Peelu* (*Salvadora persica* L) and *Sharkara* (*Saccharum officinarum* L) plants. Many of these medicinal plants have been individually reported to exhibit diverse pharmacological actions such as antiinflammatory, analgesic, hepato-protective, antimicrobial and antiulcer properties as detailed in Table 1.[2-12] Analysis of the chemical constituents of these plants revealed the presence of tannins, triterpenoids, phenols, glycosides, sucrose, glucose, flavonoids and flavonidic glycosides as enlisted in Table 1.[13-17]

**Table 1:** Pharmacological properties and chemical constituents of *Jwarhar mahakashay* drugs

Name	Scientific name	Family	Parts used	Pharmacological properties Reported	Active chemical constituents
Sariva	*Hemidesmus indicus R. Br*.	Asclepiadaceae	Roots	Anti-infl ammatory, Anti-ulcer	Flavonoids, Triterpenoids, Tannin, Phytosterol,β-sitosterol
Sharkara	*Saccharum offi cinarum* L.	Poaceae	Granules	Anti-infl ammatory, analgesic	Flavonoids, Glucosides, Glycans, Glucose, Sucrose, Phenols
Patha	*Cissampelos pareira* L.	Menispermaceae	Roots	Tumour-inhibitor	Alkaloids, Berberin, Hayatine, Cissampareine
Manjistha	*Rubia cordifolia* L.	Rubiaceae	Fruits	Anti-infl ammatory, antibacterial	Glycosides, Anthraquinone, Rubiadin, Triterpenes
Draksha	*Vitis vinifera* L.	Vitaceae	Fruits	Anti-ulcer, hepatoprotective	Flavonoids, Glucose, Fructose, Glycosides, Polyphenols
Peelu	*Salvadora persica* L.	Salvadoraceae	Fruits	Anti-ulcer, anti-microbial	Tannin, Salvadorin, Glucose, Fructose
Parushak	*Grewia asiatica* L.	Tiliaceae	Fruits	Anti-malarial, anti-ulcer	Flavonoids, Tannin, Glucose, Glycosides
Haritaki	*Terminalia chebula* Retz.	Combretaceae	Fruits	Anti-microbial, purgative	Tannin, Triterpenes, Chebulinic acid, Glycosides
Vibhitak	*Terminalia bellirica* Roxb.	Combretaceae	Fruits	Hepato-protective, antihistaminic	Gallic acid, Glycosides, Chebulagic acid, Triterpenoids
Amala	*Emblica offi cinalis* Gaertn.	Euphorbiaceae	Fruits	Anti-ulcer, anti-infl ammatory, hepato-protective	Vitamin C, Elagic acid, Polyphenol, Glucose, Phyllembin

We have already reported that the preliminary phytochemical analysis of the aqueous extract of a homogeneous mixture of the abovementioned plants prepared as laid down in Chikitsa Sthana of Charak Samhita (referred hereinafter as the research drug) had revealed the presence of tannins, reducing sugars, flavonoids, glycosides and salicylates.[18] During experimental study on rodents, this aqueous extract exhibited significant antipyretic–analgesic properties compared to the standard NSAIDS antipyretics such as Aspirin. It also exhibited significantly low ulcerogenicity and very low toxicity even at very high dosage compared to Aspirin.[18]

The present work aimed to perform a detailed chemical examination of the aqueous extract of *Jwarhar mahakashay* preparation and to identify pure chemical marker compounds in it using chromatographic and spectroscopy methods.

## MATERIALS AND METHODS

### General experimental procedures

UV-Visible spectroscopy scans and absorbance studies for assessing total polyphenol content were performed in Shimadzu UV-VIS-2550 model Spectrophotometer. Fourier transform infra-red spectroscopy (FTIR) studies were conducted in transmittance mode using FT/IR-670 Plus – JASCO model with DLATGS detector. The LC-6A Shimadzu system with UV-Visible spectroscopic detector and C_18_ column was used for high performance liquid chromatography (HPLC) analysis of the extract samples. Gas chromatography and mass spectroscopy (GC-MS) systematic evaluation was carried out in Shimadzu QP 5050 GC Mass and GC-17A Gas Chromatograph.

### Plant materials

The roots of Sariva (*Hemidesmus indicus* R. Br.), *Manjistha*(*Rubia cordifolia* L.) and *Patha* (*Cissampelos pareira* L.), fruits of *Haritaki* (*Terminalia chebula* Retz.), *Amla* (*Phyllanthus emblica* Gaertn.), *Vibhitak* (*Terminalia bellirica* Roxb.), *Draksha* (*Vitis vinifera* L..), *Parushak* (*Grewia asiatica* L..) and *Peelu* (*Salvadora persica* L..), and *Sharkara granules* (*Saccharum officinarum* L.) were obtained from the Apothecary department of the Institute of Post-Graduate Ayurvedic Education and Research, Kolkata, during May 2006. These were authenticated and identified by the Department of Ethnobotany, Botanical Survey of India, Shibpur, Howrah, and a voucher specimen was deposited in the herbarium before their utilization. Plant parts were shade dried and coarsely powdered up to 40 mesh size. Equal portions by weight of all ingredients, as mentioned in Chikitsa Sthana of Charak Samhita for treatment of pyrexia, were homogenously mixed and subjected to Soxhlet extraction in refluxing distilled water.

The extraction was continued for 48 h using distilled water four times by weight of the crude drug mixture. The aqueous extract was filtered through calico cloth and was further concentrated to a semi-solid substance under reduced pressure in a rotary evaporator, which was then dried over water bath into a solid substance.

The extract yield from 300 g of plant powder mixture was 75 g. This *Jwarhar mahakashay* preparation was used for all systematic evaluation studies and preserved at the Division of Pharmaceutical Technology Laboratory, Department of Chemical Technology, Calcutta University, Kolkata, where the studies were carried out during 2006–2007.

### Elemental analysis

Elemental analysis was performed to detect the presence of nitrogen, sulfur and halogens using routine chemical analysis techniques. A piece of metallic sodium was taken in a test tube, melted by slow heating and about 0.5 g of research drug was added and strongly heated for about 2 min. Twenty milliliter of distilled water was taken in a mortar and pastel, the red-hot test tube was broken and ground in mortar distilled water. The aqueous solution was filtered through Watman-40 filter paper and the filtrate was subjected to test for these elements.[19]

### Analysis of phytochemical constituents

Systematic analysis using standard methods was done for ascertaining the presence of different phytochemical constituents such as alkaloids, amino acids, reducing sugars, tannins, saponins, anthraquinones, steroids, terpenoids, flavonoids and salicylates.[19]

### Estimation of total polyphenol content

Total polyphenol content was estimated using the Folin–Ciocalteu method calibrated on Gallic acid.[20] Sample extracts of 500 µL were added to 500 µL of water, 5 mL of 0.2 N Folin–Ciocalteu reagent and 4 mL of 75 g /L^∂1^ saturated sodium carbonate solution and mixed in a cyclomixer. The absorbance was measured in the spectrophotometer at 765 nm after incubation for 2 h at room temperature. Quantification of total polyphenol content was done on the basis of a standard curve generated with 100, 200, 300 and 400 mg/L^∂1^ of Gallic acid.

### Hydrolysis of research drug

The phytochemical tests on *Jwarhar mahakashay* preparation were strongly indicative of the presence of flavonoids, glycosides and sugars.[18] Since flavonoids normally exhibit antipyretic, analgesic and antiinflammatory properties, systematic and standard hydrolysis procedure was followed on the research drug for detailed investigation of its chemical constituents, especially flavonoids and glycosides. A 0.25 mg of research drug was dissolved in 0.3 mL – 2N HCl : MeOH (1 : 1 v/v), sealed in a screw-cap polypropylene tube and heated on a steam bath for 30 min. The mixture was extracted with equal volume of ethyl acetate; the upper organic layer was separately collected and was subsequently evaporated to dryness under reduced pressure.[21] The residue was dissolved in methanol and was simultaneously analysed in UV-Visible spectroscopy, thin layer chromatography (TLC), HPLC, FTIR and GC Mass Spectroscopy. The aqueous layer was analysed for sugar using Fehling's solution, which was found positive.

### UV-visible spectroscopic scanning

The hydrolyzed sample of research drug was dissolved in (2% w/v) HPLC water, filtered and scanned in the spectrophotometer at medium speed in UV-Visible range for specific absorbance identification that might appear for its chemical constituents.[22] The characteristics of molecules to absorb radiations under specific wavelengths were scanned in the entire range of 190 to 800 nm. All prepared batches of research drug were routinely checked to match these UV absorbance band criterions for routine standardization purposes.

### Fourier transform infra-red spectroscopy

FTIR scan of herbal medicaments provide for quality finger printing for identifiable absorbance bands due to presence of individual functional groups. Hydrolyzed sample of research drug was dried in rotary evaporator, mixed with KBr, palletized and subjected to FTIR studies in transmittance mode.[23]

### Chromatography analysis

Different chromatography techniques such as TLC, HPLC, GC-Mass, etc. were used for evaluation and component standardization of hydrolyzed sample research drug.

### Thin layer chromatography

The research drug was analysed using a glass plate coated with a thin layer of silica gel (194015 G, SISCO Research laboratories Pvt. Ltd., Mumbai, India) using different solvent mixtures. The different spots developed were visualized on coloration (like Iodine vapor exposure) and their *R_f_* values were calculated.*R_f_* values of components are indicative of specific character of molecule in the given environment of mobile and stationary phase. Best results were obtained with an ethyl acetate/butane/formic acid/water (30 : 20 : 10 : 40 v/v/v/v) solvent system.

### High performance liquid chromatography

The HPLC flow rate was 0.5 ml/min using methanol/water (70 : 30) as the mobile phase solvent, under a pressure of 100 kgf/sq.cm, run time of 20 min and an injection volume of 20 µL. Analysis was performed at 220 nm and repeated at 276 nm, which are the two points where absorbance maxima were observed during the UV-Visible spectroscopy.

### Gas chromatography and mass spectroscopy

For exact identification of principle components present in the research drug as chemical marker, systematic GC-MS evaluation was carried out using freshly prepared ethyl acetate extract of flavonidic hydrolyzed sample. Ethyl acetate extract was initially evaporated under reduced pressure at room temperature and the residual concentrate re-dissolved in HPLC methanol. Both injection temperature and interface temperature were maintained at 250° C, while the split ratio was set at 1 : 20. The flow rate of Helium in the column (ZB5 type, diameter 0.25 mm, length 30 m) was 0.6 mL/min and the carrier flow rate was 13 mL/min. The column temperature was initially set at 45°C and thereafter increased at the rate of 15°C/min till it reached 250°C. Mass spectroscopy was done in the range of 45 to 350 m/z and data were captured at time intervals of 0.5 s.[24]

## RESULTS

### Elemental analysis

Only sulfur was found present in research dry, while nitrogen and halogens were found to be absent.

### Analysis of phytochemical constituents

Flavonoids, reducing sugar, tannins, glycosides and salicylates were found present, while alkaloids, steroids, terpenoids, amino acids, anthraquinones and saponins were found to be absent during analysis of its phytochemical constituents.

### Estimation of total polyphenol content

The total polyphenol content in 1 mg of test drug was estimated to be 0.0535 mg of Gallic Acid Equivalents using the absorbance calibration curve generated with different concentrations of Gallic acid. Thus, the overall polyphenol content in *Jwarhar mahakashay* preparation was assessed as 5.35% (w/w) using the Folin–Ciocalteu method.

### UV-visible spectroscopic scanning

**Figure 1:**
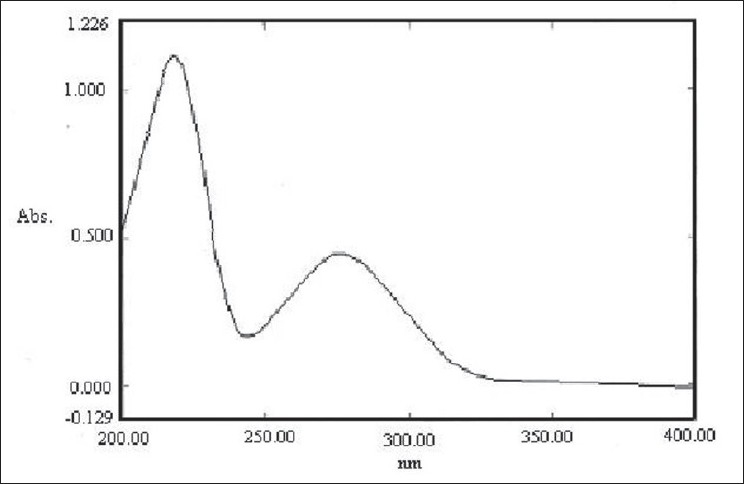
UV-visible spectroscopic scan of research drug

The characteristics of molecules to absorb radiations under specific wavelengths were scanned in the entire range of 190 to 800 nm. The UV-Visible spectroscopic scan of the hydrolyzed sample is presented in Figure 1. Two absorbance maxima were observed at 276 and 220 nm.

### Fourier transform infra-red spectroscopy

**Figure 2:**
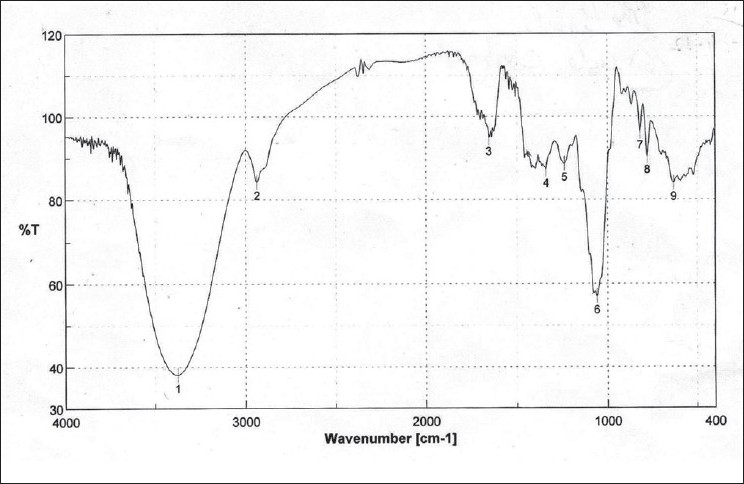
FTIR finger printing of research drug

Fourier Transform Infra-red Spectroscopy (FTIR) study of dry-powdered test drug in KBr pellet was investigated to identify the presence of functional groups. FTIR scan does not corroborate the presence of any nitrogenous functional group like -NH, -NHR, -NO, NO_2_ etc. However, characteristic broad alcoholic -OH in hydrogen-bonded structure was observed at 3360/cm. Such alcoholic-OH was confirmed for the presence of C–O stretching vibration at 1065/cm. Tautomeric C=O stretching was observed for aldehydic presence at 1647/cm. The FTIR results were presented in Figure 2. This end carboxylic O–H presence provided at 2940/cm is confirmatory to original chemical investigation for presence of glycoside-linked tannins or flavonoids.

### Thin layer chromatography

After several runs in different solvent environments, flavonoids in hydrolyzate moved distinctly in flavonoid-specific solvent front ethyl acetate : butanone : formic acid : water (30 : 20 : 10 : 40) with *R_f_* values of 0.344, 0.7444 and 0.967. Preliminary separation in TLC is suggestive of more than one flavonidic components.

### High performance liquid chromatography

**Figure 3:**
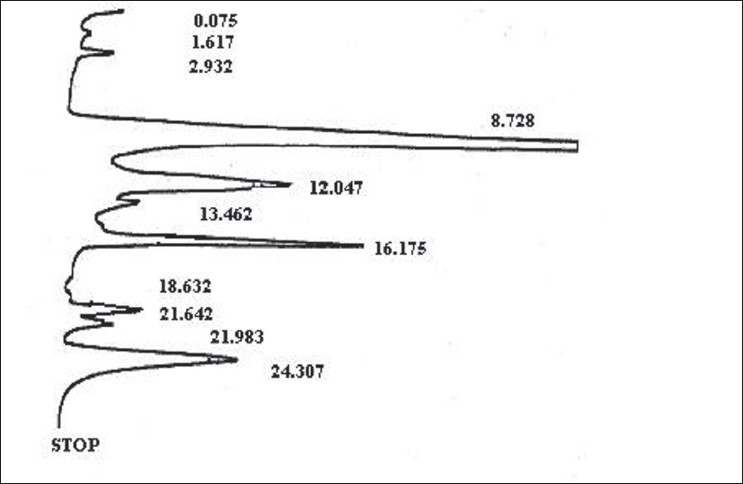
HPLC chromatogram of research drug at 220 nm

**Figure 4:**
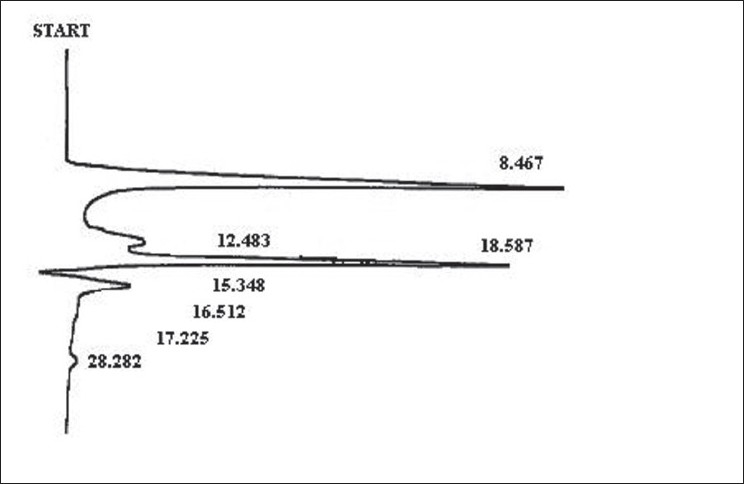
HPLC chromatogram of research drug at 276 nm

The HPLC analysis at 220 nm wavelength provided the majority component eluting at 8.7 min, while minor peaks appeared at 12.1, 16.2 and 24.3 min. Similarly, running HPLC finger printing at 276 nm provided a distinct pattern with the majority components appearing at 8.5 and 13.6 min. The results of HPLC analysis have been presented in Figures 3 and 4.

### Gas chromatography and mass spectroscopy

**Figure 5:**
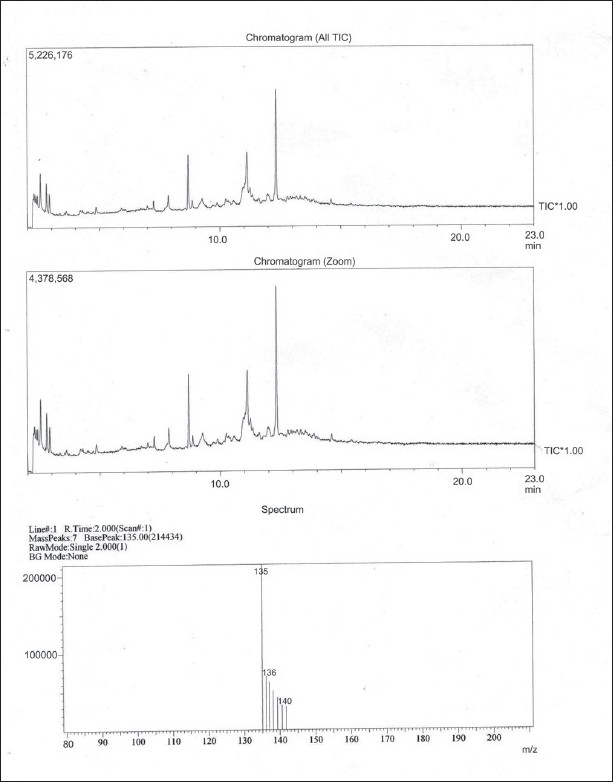
Results of GC–Mass spectroscopy of research drug

**Figure 6:**
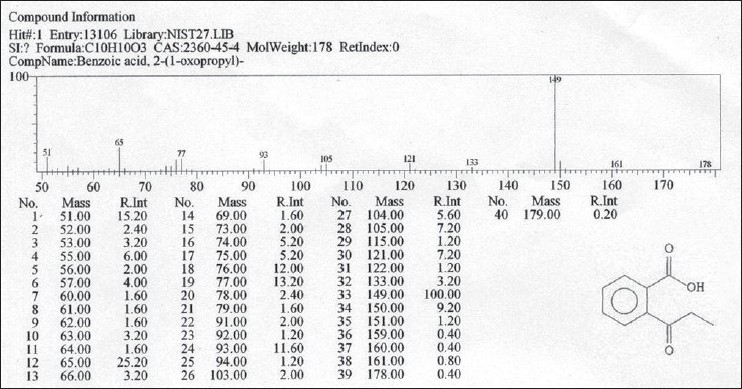
Identification of the majority component in research drug using GC–Mass analysis

The results of the GC–Mass Spectroscopy analysis are presented in Figures 5 and 6. Detailed analysis of concentrate provided one major and two minor components that were corroborated when chromatogram was zoomed. Detailed analysis of the two minor components was not performed since their relative intensity was found to be quite low (25.2 and 13.2%) as compared to the majority component (100%) as detailed in Figures 5 and 6. The majority component could be identified in comparison to Nist and Willey library as 2-(1-oxopropyl)-benzoic acid. This, considering all structural possibilities, could be one of the components that provided antipyretic–analgesic activity of the research drug since it is quite similar to the active ingredient 2-acetyl-oxybenzoic acid contained in Aspirin (acetylsalicylic acid), a common nonsteroidal antipyretic formulation.

## DISCUSSION

Detailed chemical investigations were undertaken to analyse the basic chemical nature, to establish uniformity in quality standard of the research drug and to identify pure chemical marker compounds in it. Elemental analysis confirmed the presence of sulfur, while nitrogen and halogens were found to be absent. Preliminary phytochemical screening of the extract indicated the presence of tannins, reducing sugars, flavonoids, glycosides and salicylates.[18] Of these, flavonoids are well known for their ability to inhibit pain perception[25] and to exhibit antiinflammatory properties due to their inhibitory effects on enzymes involved in production of the chemical mediator of inflammation.[27] Flavonoids and its related compounds also exhibit inhibition of arachidonic acid peroxidation, which results in reduction of prostaglandin levels thus reducing the fever.[27] Since flavonoids exhibit several biological effects such as antiinflammatory, antimicrobial, antihepatotoxic and antiulcer activities,[2,28] it is likely that the antipyretic action of *Jwarhar mahakashay* preparation is primarily related to the presence of flavonoids. Therefore, standard hydrolysis procedure was followed for detailed investigation of its chemical constituents, especially flavonoids and glycosides, by using UV-Visible spectroscopy, TLC, HPLC, FTIR and GC–Mass Spectroscopy.

During UV-Visible spectroscopic scanning, absorbance maxima were observed at 276 and 220 nm. The FTIR scan indicated an end carboxylic O–H structure at 2940 cm^−1^ , which confirms the findings of the phytochemical investigation regarding the presence of glycoside-linked tannins or flavonoids in the research drug.

TLC and HPLC studies were performed for standardization and preliminary identification of the chemical parameter constituents in the research drug. Preliminary separation in Thin Layer Chromatography was suggestive of more than one flavonidic components. HPLC analysis at 220 and 276 nm wavelength exhibited three majority components eluting around 8, 12 and 13 min as represented by the local peaks in the resultant graphs.

Gas Chromatography–Mass Spectroscopy was finally done for exact identification of principle components present in *Jwarhar mahakashay* preparation as chemical marker by systematic evaluation of ethyl acetate extract of the sample. Detailed analysis of concentrate provided three major components that were corroborated when chromatogram was zoomed and the majority component could be identified in comparison to Nist and Willey library as 2-(1-oxopropyl)-benzoic acid.

The chemical analysis of the *Jwarhar mahakashay* preparation in order to ascertain the marker compounds indicated the presence of flavonoids, specifically the glycosidic flavonoids, which are well known for their antipyretic properties. Detailed analysis revealed the presence of at least one active compound, which is structurally quite similar to the active constituent of a well-known NSAIDs compound, the Aspirin.
